# RluA is the major mRNA pseudouridine synthase in *Escherichia coli*

**DOI:** 10.1371/journal.pgen.1011100

**Published:** 2024-09-06

**Authors:** Cassandra Schaening-Burgos, Hannah LeBlanc, Christian Fagre, Gene-Wei Li, Wendy V. Gilbert

**Affiliations:** 1 Department of Biology, Massachusetts Institute of Technology; Cambridge, Massachusetts, United States of America; 2 Program in Computational and Systems Biology, Massachusetts Institute of Technology, Cambridge, Massachusetts, United States of America; 3 Department of Molecular Biophysics and Biochemistry, Yale University, New Haven, Connecticut, United States of America; Friedrich-Schiller-Universitat Jena, GERMANY

## Abstract

Pseudouridine (Ψ) is an ubiquitous RNA modification, present in the tRNAs and rRNAs of species across all domains of life. Conserved pseudouridine synthases modify the mRNAs of diverse eukaryotes, but the modification has yet to be identified in bacterial mRNAs. Here, we report the discovery of pseudouridines in mRNA from *E*. *coli*. By testing the mRNA modification capacity of all 11 known pseudouridine synthases, we identify RluA as the predominant mRNA-modifying enzyme. RluA, a known tRNA and 23S rRNA pseudouridine synthase, modifies at least 31 of the 44 high-confidence sites we identified in *E*. *coli* mRNAs. Using RNA structure probing data to inform secondary structures, we show that the target sites of RluA occur in a common sequence and structural motif comprised of a ΨURAA sequence located in the loop of a short hairpin. This recognition element is shared with previously identified target sites of RluA in tRNAs and rRNA. Overall, our work identifies pseudouridine in key mRNAs and suggests the capacity of Ψ to regulate the transcripts that contain it.

## Author summary

While RNAs are initially transcribed using only the nucleotides A, G, C and U, these nucleotides can be enzymatically modified into many different post-transcriptional modifications. tRNAs and rRNAs across all domains of life are modified with pseudouridine, an isoform of uridine that is inserted by highly conserved enzymes. In many eukaryotes, it has been demonstrated that some of these enzymes can also insert pseudouridines in mRNA, where they are poised to impact gene expression through their effects on the transcript. Here we show that protein-coding transcripts in *E*. *coli* are also modified with pseudouridine, indicating that mRNA pseudouridylation is also a highly conserved activity. RluA is the main mRNA pseudouridine synthase, introducing the modification into the transcripts of dozens of protein coding genes with high specificity. Its target sites are defined by a combined sequence and secondary structure motif. Two additional enzymes, RluC and RluD, introduce a few additional sites. All three of these enzymes belong to the same sub-family of pseudouridine synthases, and homologs of these also modify mRNAs in humans. Thus, mRNA modification by these enzymes might be a conserved activity with the capacity to impact gene regulation.

## Introduction

All organisms modify their tRNAs and rRNAs extensively, introducing dozens of unique RNA modifications in many positions. It has become clear that mRNAs are also modified, as evidenced by the discovery of at least nine distinct modifications in the mRNAs of organisms across all domains of life [1–10]. Pseudouridine (Ψ) in particular appears especially widespread, having been identified in the mRNAs of human, mice, yeast, *Trypanosoma brucei*, and *Arabidopsis thaliana* [1,2,11,12]. However, it has yet to be detected in bacterial mRNAs.

**Fig 1:**
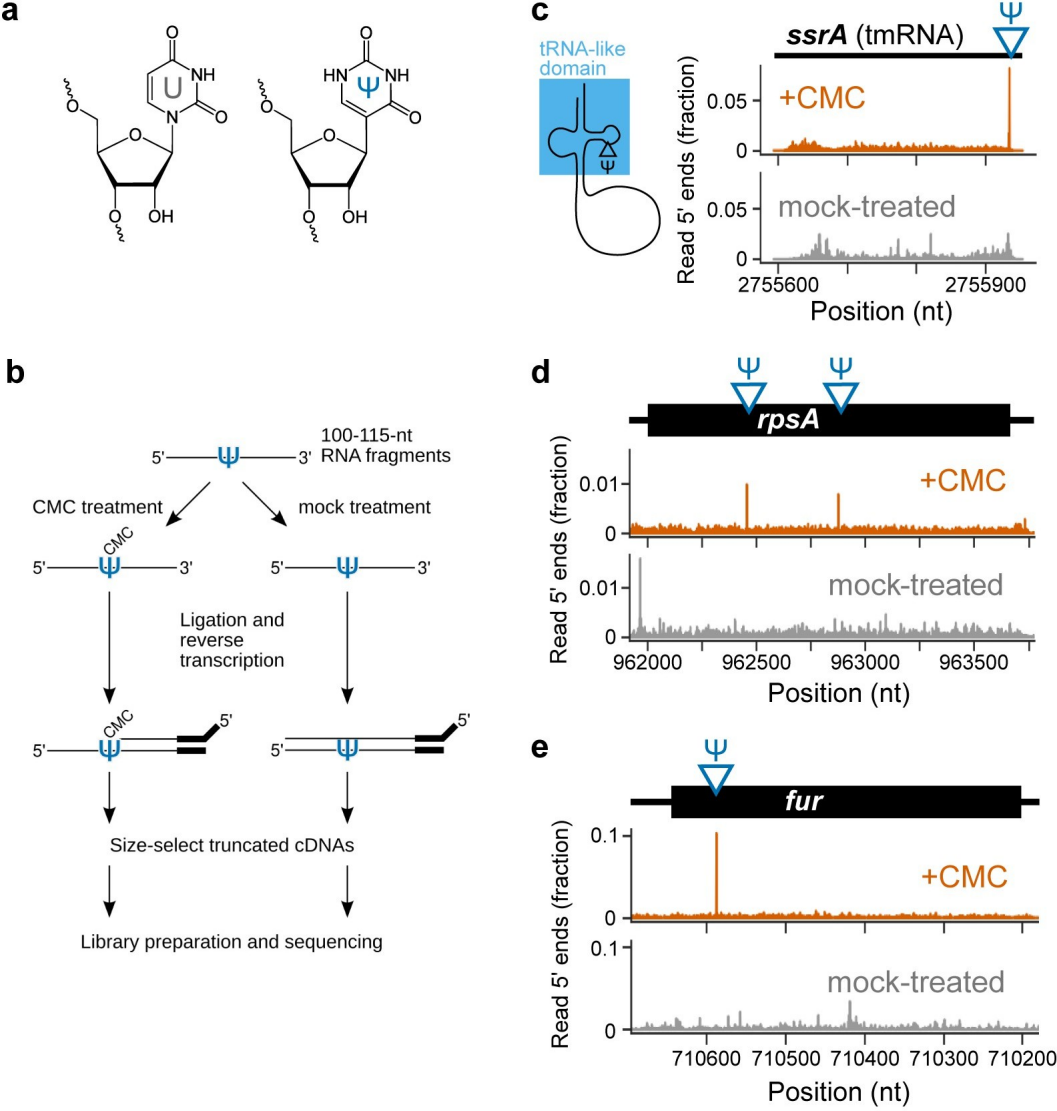
Pseudo-Seq identifies known and novel pseudouridine sites in the *E*.*coli* transcriptome. (a) Structures of uridine and pseudouridine. (b) Diagram of the Pseudo-Seq method. Treatment with CMC causes robust reverse transcription (RT) stops at pseudouridine sites. These truncated cDNAs are enriched via size selection and captured for sequencing. (c) Validation of a known site in the tmRNA gene, *ssrA*. Its tRNA-like domain contains a pseudouridine, indicated on the gene track and secondary structure diagram. Data tracks show read 5′ ends, normalized to total transcript reads, in wild-type CMC-treated samples (orange) and mock-treated samples (gray). (d) Pseudo-seq signal for two novel mRNA sites on the *rpsA* mRNA and for (e) a novel site in the *fur* mRNA.

Pseudouridine is an isomer of uridine, generated by rotating the uridine base to obtain an additional hydrogen bond donor and replacing the C-N glycosidic linkage with a C-C bond, while maintaining the same Watson-Crick face (Fig 1A) [13]. Pseudouridylation is carried out by a universally conserved class of enzymes called pseudouridine synthases, which insert the modification in tRNAs and rRNAs, as well as other non-coding RNAs [14,15]. In eukaryotes, some of the same enzymes that modify these canonical targets also insert pseudouridines into mRNAs [2,16,17]. Because the pseudouridine synthases in *E*. *coli* belong to this conserved family, it is plausible that their mRNAs are also modified with Ψ.

Pseudouridylation of rRNAs and tRNAs in *E*. *coli* is carried out by eleven pseudouridine synthases [15]. Four of the rRNA:Ψ synthases (RluB, RluE, RluF, and RsuA) modify single positions in the ribosomal RNA, whereas the tRNA-modifying synthase TruB modifies Ψ55 in most tRNAs. The synthases RluA, RluC, and RluD are notable in their ability to modify sites in more diverse contexts. RluA has been described as a multi-site and multi-substrate enzyme for its ability to specifically modify Ψ32 in four tRNAs as well as a single position in the large subunit rRNA [18]. RluC and RluD are both multi-site enzymes that each modify three different positions in rRNA: RluD modifies three sites in helix 69 of the large subunit rRNA (Ψ1911, Ψ1915, which is further modified into m^3^Ψ by an additional enzyme, and Ψ1917), while RluC modifies Ψ955, Ψ2504 and Ψ2580 [19]. It is currently unknown whether these enzymes also modify mRNAs.

Through its biochemical effects, Ψ is poised to affect multiple stages in the life cycle of an mRNA. Pseudouridine has been consistently shown to stabilize RNA duplexes, including secondary structures and intermolecular RNA:RNA interactions [20–22]. Through this mechanism, pseudouridine has the potential to modulate regulatory structures in mRNA, such as riboswitches, as well as interactions with small regulatory RNAs. Ψ can also impact the conformation of unpaired RNA regions by enhancing base stacking [23].

Pseudouridine can inhibit RNA-protein interactions by conferring additional backbone rigidity. *In vitro*, this has been demonstrated with pseudouridylated oligos and at least three different RNA-binding proteins, each of which had reduced affinity for pseudouridylated RNA compared to unmodified RNA [24–26]. Similarly, modification can affect the activity of enzymes acting on RNA; recent work showed that the ribonuclease RNase E cleaves pseudouridylated RNAs less efficiently than unmodified ones, and did so at altered positions [27]. Lastly, there is growing evidence for effects on translation fidelity: pseudouridine in stop codons can allow for stop-codon readthrough [28], whereas pseudouridine in some sense codons has been shown to enable translational recoding [29]. This variety of effects highlights an important principle: the impact of pseudouridine on a given mRNA is likely to be highly context-dependent and subject to adaptive evolution, based on its interactome and local sequence and structure.

Here, we extensively characterize the pseudouridylation profile of exponential-phase *E*. *coli*, and determine the mRNA modification capacity of each of its 11 pseudouridine synthases. We discovered that mRNA pseudouridylation is carried out predominantly by the synthase RluA, posing it as a novel regulator of mRNA processing and function. This enzyme introduces pseudouridines in at least 31 mRNA sites and is likely to modify additional sites that are lowly expressed. We characterize the sequence and structural context of the sites modified by RluA and determine that they occur in a common ΨURAA motif, often positioned within a small secondary structure element which resembles the ribosomal target site of RluA.

## Results

### Mapping pseudouridines by Pseudo-Seq

Pseudouridine detection can be enabled by chemical treatment that generates a modification-dependent signature in sequencing datasets, even though Ψ by itself cannot be detected through reverse transcription and sequencing. One such reagent is CMC (N-cyclohexyl-N′-β-(4-methylmorpholinium)ethylcarbodiimide p-tosylate), a bulky carbodiimide that derivatizes the NH groups in Ψ and causes robust termination during reverse transcription [30]. To detect pseudouridines in the *E*. *coli* transcriptome, we used the Pseudo-Seq method [1], which combines CMC treatment with size-selection steps that enrich truncated reverse transcription products, followed by high-throughput sequencing (Fig 1B). This method generates a CMC-dependent pileup of reads whose 5′ ends are immediately downstream of the modified site (Fig 1C). A mock-treated sample is prepared in parallel to remove potential false positives from other types of events that can generate reverse transcription stops, such as strong secondary structure, other RNA modifications, or transcript ends. These events appear as CMC-independent peaks, so CMC dependence is a requirement for calling a peak as a pseudouridine.

We applied Pseudo-Seq to wild-type *E*. *coli* grown to exponential phase (OD_600_ ∼ 0.10–0.15). This approach successfully reproduced a known site in the tRNA-like domain of tmRNA (Fig 1C) [31], as well as several known sites in tRNAs and rRNAs (S1 Fig). Each of these sites exhibits the expected CMC-dependent peak at the annotated position, indicating that known sites are reliably captured in our dataset.

### *E*. *coli* mRNAs are abundantly modified with pseudouridine

**Fig 2:**
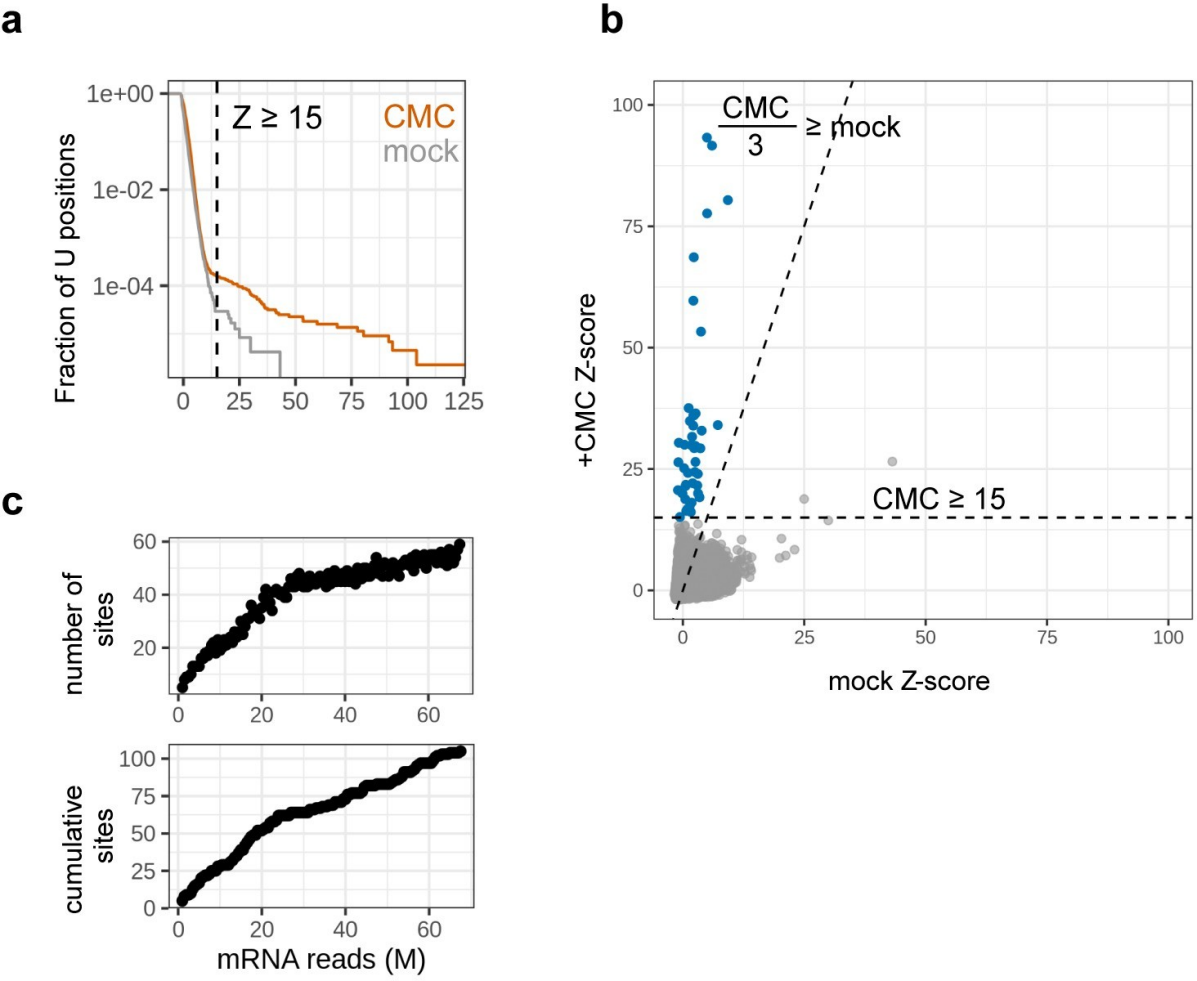
Systematically calling pseudouridine sites transcriptome-wide. (a) Z-score distribution for all uridines in mRNA in wild-type libraries, with CMC-treated in orange and mock-treated in gray. Distribution is visualized as a complementary cumulative density function, indicating the fraction of positions that have a Z-score greater than a given value. The cutoff for calling a peak in the CMC-treated library, Z ≥ 15, is indicated by the dashed line. (b) Joint distribution of mRNA Z-scores in CMC-treated (y-axis) and mock-treated (x-axis) libraries. Cutoffs for calling potential pseudouridine sites are indicated by the dashed lines: sites must have a Z-score ≥ 15 in the CMC-treated library, and must be at least 3-fold greater than the corresponding peak in the mock-treated library. Sites that meet both cutoffs are considered potential pseudouridine sites and are shown in blue. (c) Number of CMC-dependent peaks called at increasing mRNA read coverage. Reads from multiple wild-type samples were pooled to achieve the top coverage shown, and reads were subsampled from this pool to read depths ranging from 300,000 to 36 million mRNA-mapping reads. Top panel shows the number of peaks called in each subsample, bottom panel shows the cumulative number of unique peaks.

To identify pseudouridines in *E*. *coli* mRNAs along with the responsible pseudouridine synthases, we generated Pseudo-Seq datasets for wild-type *E*. *coli* and for individual knockout strains of all 11 pseudouridine synthases. In the wild-type dataset, we observed strong CMC-dependent peaks in some mRNAs, indicating the presence of pseudouridines in protein-coding genes (Fig 1D and 1E). In order to systematically call Ψ sites transcriptome-wide while accounting for biases arising from nearby modifications or transcript ends, we implemented a modified Z-score that includes 95% winsorization and background window adjustment (S2 Fig, Methods). We identified candidate pseudouridine sites as strong peaks in the CMC-treated library that were absent from the mock-treated library. Based on the distribution of Z-scores among uridines in mRNAs, we defined these strong peaks as those that have a Z-score greater than 15 (Fig 2A) and at least 3-fold greater than the Z-score in the mock-treated library (Fig 2B). Using these stringent criteria, we were able to validate 8 out of the 10 ribosomal RNA sites, with no false positives (S2C Fig).

We then applied the modified Z-score metric to reads mapped to the entire *E*. *coli* transcriptome (Fig 2B, Methods). Peak height calculations are very noisy at lower sequencing coverage (S2D Fig), necessitating strict coverage cutoffs in the background region surrounding each putative site. Applying these cutoffs limits site calling to genes with sufficient expression, and so the number of sites called in a library will vary depending on sequencing depth. To address this issue, we generated a high-depth wild-type Pseudo-Seq library, which spans 24.2% of uridines in protein-coding genes, to call a high-confidence list of sites. The profiled uridines encompass 1532 protein-coding genes, out of the 4099 genes detected in our sequencing data. The genes sequenced to sufficient depth for pseudouridine detection were expressed to a median RPKM of 201, while the overall median expression level was 44.4 (S2E Fig).

This top-tier list of targets consisted of 44 mRNA sites (S1 Table). 43 of these were within coding-sequences, where they could potentially impact the fidelity and rate of translation. Modifications outside of the coding sequence were comparatively rare, as expected from the relative sizes of these regions in *E*. *coli*, with only one modification in the 5′ UTR, and none in the 3′ UTR.

The genes modified with pseudouridine do not appear to form a coherent regulon—rather than targeting genes that are part of the same processes or pathways, the modification is present in mRNAs encoding components of many different biological processes. Among these, there is modification of genes that encode proteins involved in translation, including three ribosomal proteins (RpsA, RpsH, RpsQ), two translation elongation factors (TufA, FusA), and one tRNA biogenesis factor (Fmt) (S1 Table).

Sequence coverage is the limiting factor in calling new sites. Thus, most called sites are in highly expressed genes. To determine whether pseudouridylation also occurs in mRNAs with lower expression levels, we simulated higher coverage by pooling reads from different Pseudo-Seq libraries, and subsampling the pooled libraries to depths ranging from 1 million to 67.5 million mRNA reads. Increasing coverage in this manner identified many new CMC-dependent peaks, with up to 100 sites detected when coverage exceeds 65 million mRNA-mapping reads, indicating that pseudouridine is also present in genes with lower expression levels. (Fig 2C and S2 Table).

### RluA is the predominant mRNA pseudouridylation enzyme

Pseudouridine is installed in *E*. *coli* tRNAs and rRNAs by a universally conserved class of pseudouridine synthases. These enzymes are derived from an ancestral pseudouridine synthase, identified by a common fold, and belong to five different families (with an additional sixth family in eukaryotes) [15,32]. Each of these five families is represented among the *E*. *coli* pseudouridine synthases, with two of these families having diversified further, for a total of 11 pseudouridine synthases. To determine which of these enzymes carry out mRNA pseudouridylation, we obtained knockout strains of each of these synthases and carried out Pseudo-Seq on cultures grown to exponential phase (OD_600_ ∼ 0.10–0.15).

**Fig 3:**
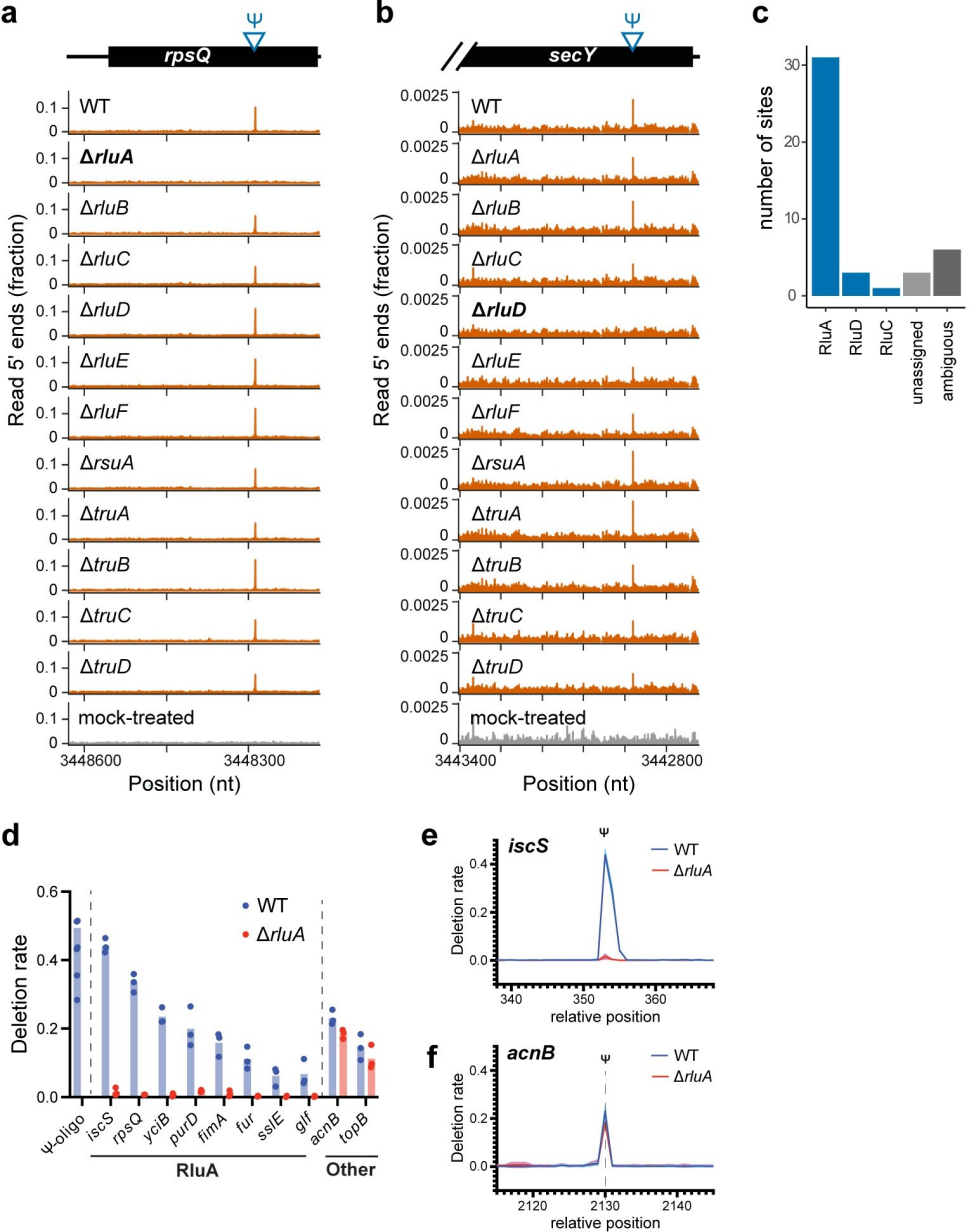
RluA is the predominant mRNA pseudouridine synthase in *E*. *coli*. (a) Pseudo-Seq signal for a sample mRNA site in 13 libraries: wild-type, all 11 pseudouridine synthase knockouts, and a mock-treated control for the wild-type. The site appears as an RluA- and CMC-dependent peak in read 5′ ends. (b) Pseudo-Seq signal for a sample RluD-dependent site in an mRNA. The site appears as a RluD- and CMC-dependent peak in read 5′ ends. (c) Enzyme assignment for the highest confidence sites. In order to assign enzymes, we required sufficient coverage in both the wild-type and knockout libraries, and for the peak to be both CMC- and synthase-dependent. (d-f) Targeted bisulfite sequencing of select Ψ sites identified by Pseudo-Seq. (d) Deletion rate at Ψ position at 10 selected sites in WT and Δ*rluA* strains. “Ψ-oligo” is a pool of synthetic RNA installed with 100% Ψ within variable sequence contexts, as a reference for the magnitude and spread of deletion signal given complete modification. (e-f) Trace of deletion signature at candidate sites in WT and Δ*rluA* strains. (e) is an RluA-dependent site in *iscS*, and (f) is an unassigned RluA-independent site in *acnB*.

Enzyme-dependent sites are expected to show a CMC-dependent peak across all libraries except the relevant knockout (Fig 3A and 3B, Methods). In this manner, 31 out of the 44 high-confidence sites are unambiguously assigned to RluA. There is additional modification carried out by the enzymes RluC and RluD (Fig 3C); RluD unambiguously modifies at least three mRNA sites, while RluC modifies a single site in the *atpG* mRNA. Each of these three enzymes is known to modify multiple sites among tRNAs and rRNAs. RluA modifies four tRNAs and a single position in rRNA; the RluD enzyme was previously known to modify three sites in the 23S rRNA, all in the loop of helix 69; and RluC modifies three sites in separate regions of the 23S rRNA. Meanwhile, the remaining synthases appear to modify only a single rRNA site, or a specific position in tRNAs.

To orthogonally validate high-confidence sites and estimate modification stoichiometry, we performed bisulfite sequencing [33,34] using targeted PCR amplification of selected candidate sites. Instead of forming modification-dependent cDNA truncations, bisulfite sequencing produces a 1–2 nucleotide deletion signature (Fig 3E and 3F), with a frequency roughly proportional to absolute modification rate [33]. All 10 sites selected for validation showed clear deletion signatures at modification positions identified by Pseudo-Seq (Figs 3D and S4). Comparison to synthetic RNA oligos containing 100% Ψ (Ψ-oligo) indicated several of these sites (*iscS*, *rpsQ*) had nearly total modification occupancy, while others (*sslE*, *glf*) were only sparsely modified (Fig 3D). Of these 10 sites, 8 were identified as RluA-dependent by Pseudo-Seq. Indeed, all RluA-assigned sites showed almost no deletion signature in Δ*rluA* RNA, while unassigned sites showed comparable signal to between Δ*rluA* and WT, indicating accurate and reproducible enzyme assignment.

Nine of the high-confidence pseudouridine sites could not be conclusively assigned to an enzyme. In these cases, low sequencing coverage of the target site in a subset of the libraries prevented unambiguous assignment. Sites remain unassigned (Methods) when they exhibit a strong peak in all libraries where they have high enough coverage to call a CMC-dependent peak in the wild-type library, but insufficient coverage in one or more knockouts prevents the absence of a peak from being confidently called. Meanwhile, sites can be ambiguously assigned when the peak is absent in two or more knockout libraries. This ambiguity could be biological, a consequence of partially redundant modification by more than one enzyme, or technical, resulting from low coverage.

**Fig 4:**
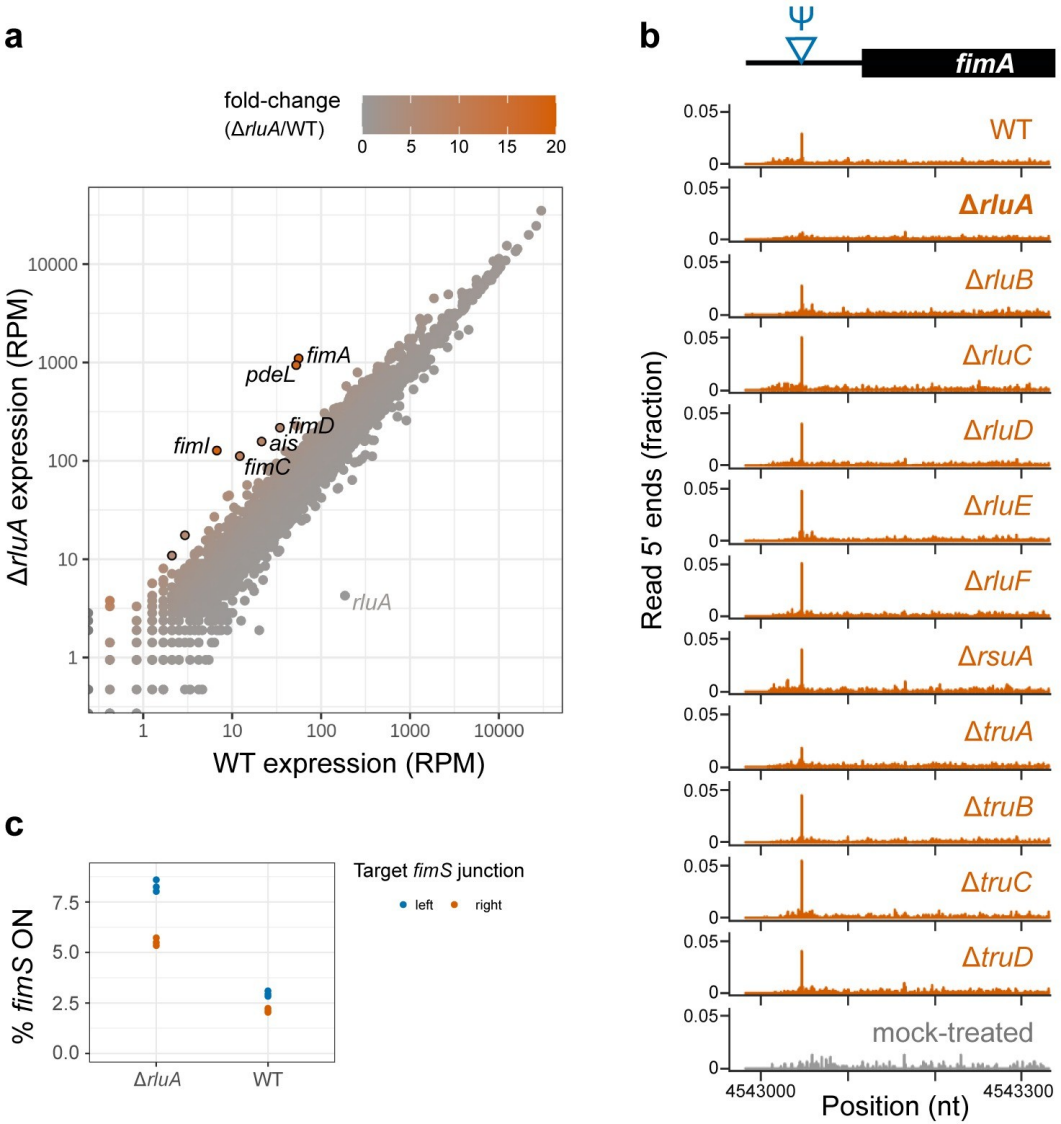
Loss of RluA alters gene expression. (a) Gene expression data in wild-type (x-axis) and Δ*rluA* (y-axis). Genes are colored by their fold-change in expression relative to wild-type. RPM: reads per million. (b) Pseudo-Seq read 5′ end counts for CMC treated libraries (orange) in every pseudouridine synthase knockout background, and in a mock treated library (gray). Counts are expressed as a fraction of reads mapped to the transcript. Read coverage contributing to *rluA*’s RPM originates from portions of the transcript left behind by the deletion, although the *rluA* protein-coding sequence is fully removed. (c) *fimS* ON conformation frequency, measured through qPCR using primers that amplify only the ON conformation. Two sets of primers were used, targeting the 5′ and 3′ junctions of *fimS*.

We performed RNA sequencing on wild-type and Δ*rluA* to examine the effects of RluA on expression. We identified few changes overall. A single mRNA target of RluA, *fimA/*the *fim* operon, was substantially affected. The 5′ UTR of the *fim* operon contains an RluA-dependent pseudouridine site (Fig 4A). The *fim* operon encodes a polycistronic transcript with the components of the Type 1 pilus, which are rod-like structures on the outer membrane of *E*. *coli* that promote surface adhesion. *fimA*, which encodes the major subunit of the pilus, experienced the greatest fold-change in expression, though all genes in the operon were upregulated. This upregulation was highly specific for the *fim* operon, as the *rluA* knockout did not result in global changes in gene expression (Fig 4A). One additional gene, *pdeL*, was substantially upregulated in Δ*rluA*, but its sequence coverage in wild-type was insufficient to determine whether it is pseudouridylated.

To determine whether the *fimA* upregulation in Δ*rluA* is due to transcriptional or post-transcriptional effects, we constructed a reporter of the *fimA* RNA driven by an exogenous promoter (S5A Fig). There was no substantial difference in the reporter expression levels between wild-type and Δ*rluA*, suggesting that RluA’s effect on *fimA* expression occurs during transcriptional regulation of the gene, rather than through post-transcriptional processes (S5B Fig). Expression of the *fim* operon can be controlled by the invertible genetic element *fimS*, which is located upstream of *fimA* [35]. *fimS* can be in an ON conformation that allows high *fim* expression, or it can be inverted into its OFF conformation. RT-qPCR measurements show that the wild-type samples were in the ON conformation with an approximate frequency of 2.5%, and the frequency increased to up to 8.5% in Δ*rluA* (Fig 4C), consistent with the higher expression observed by RNA-Seq.

### RluA modifies positions in mRNA, tRNA, and rRNA that share a sequence and structure motif

Our results illustrate the diversity of pseudouridine synthase targets, which span multiple classes of RNA. Strikingly, the enzyme RluA is capable of specifically modifying sites in tRNA, rRNA, and mRNA, despite the drastic differences in structure and life cycle among these classes. By contrast, most of the other *E*. *coli* pseudouridine synthases modify sites in a single region or position. We next show that RluA modification across these distinct sites are likely guided by a common sequence and structure motif.

**Fig 5:**
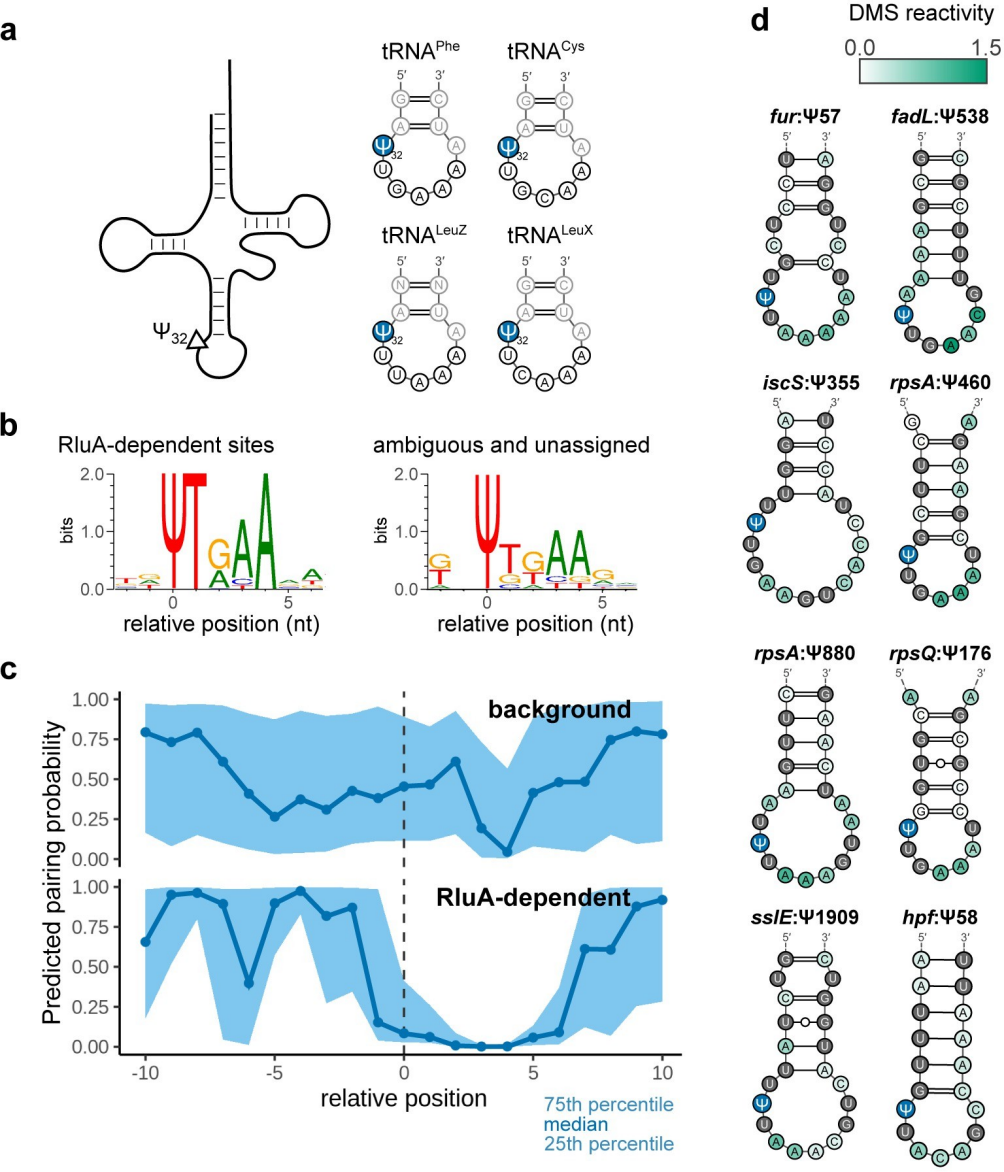
RluA targets tRNAs, rRNA, and mRNAs with a common sequence and structure motif. (a) Sequence and structure of all four canonical tRNA targets of RluA. (b) Sequence logos illustrating the sequence motif for the high confidence RluA-dependent mRNA sites (left, N = 31) and for ambiguous and unassigned sites (right, N = 9). (c) Predicted pairing probabilities for the mRNA targets of RluA. DMS-seq reactivities in the 100-nt surrounding each site were obtained from Burkhardt *et al*. (2017) [37] and used to inform secondary structure prediction. Background set consists of 94 highly expressed sites containing the UURAA motif, but lacking a CMC-dependent peak. (d) Sample structures of RluA target mRNAs. Shades of green indicate DMS reactivity, and the pseudouridine site is highlighted in blue.

RluA’s canonical targets, which consist of one site in the large subunit rRNA and position 32 in the anticodon stem-loop for four different tRNAs [18,36], occur within a ΨURAA motif (where R indicates an A or a G), in which positions U_1_ and A_4_ are particularly important for recognition (Fig 5A) [36]. We observe that the high-confidence targets of RluA occur in a similar context as the canonical sites, with highly constrained U_1_ and A_4_ positions, and a strong preference for purines at position +2 and for A at +3 (Fig 5B). This sequence motif is also enriched among the ambiguous and unassigned sites (Fig 5B), indicating that RluA is likely to modify more sites than those we have identified under our stringent hit-calling criteria.

The UURAA motif is ubiquitous in the transcriptome, with over 8000 instances sequenced to sufficient depth in this dataset, but the majority of these instances did not exhibit a CMC-dependent peak (S6 Fig). Therefore, this simple motif cannot be sufficient for modification, and additional features beyond the presence of a sequence motif must be required for recognition. The tRNA and rRNA targets of RluA share a similar structure, with the pseudouridines located in the loops of short hairpins, within one or two nucleotides of the last 5′-paired base [36]. Therefore, we sought to determine whether the mRNA targets of RluA occur in a similar structure context as the canonical sites in tRNA and rRNA. To determine the structural context of mRNA sites, we carried out dimethyl sulfate (DMS)-informed secondary structure prediction, using published RNA structure probing data from [37]. These data provide DMS reactivity scores for each A and C nucleotide, with higher reactivity indicating more accessible nucleotides. Visualizing the distribution of predicted pairing probabilities for each position relative to the Ψ, we observe that the RluA mRNA sites follow a very similar pattern as the canonical tRNA and rRNA sites, suggesting a common structural motif (Fig 5C). Inspection of the predicted structures confirms that this is the case (Figs 5D and S7).

## Discussion

Our work demonstrates that many *E*. *coli* mRNAs are modified with pseudouridine, and that these modifications are overwhelmingly carried out by the synthase RluA. Under exponential growth in rich media, we identify 44 high-confidence sites in 42 highly expressed genes, and estimate that there are at least 80 total sites in mRNAs under these growth conditions. Because Pseudo-Seq relies on reverse transcription stops and ligation to capture pseudouridine sites, our results are not quantitative, and therefore we cannot systematically infer what percentage of Us at a given position are pseudouridine. Independent validation on selected sites using BID-seq shows that some are highly modified. It will be interesting to quantitatively examine the stoichiometry of pseudouridylation across all sites through targeted mutational mapping approaches [33,38] or through direct RNA sequencing [39].

Prior to this study, very few RNA modifications had been profiled in *E*. *coli* mRNAs, and only two types of modification had been conclusively located. Adenosine-to-inosine editing has been found in 15 transcripts, where they enable recoding of tyrosine codons [9]. Meanwhile, N6-methyladenosine was found to be abundant in *E*. *coli* mRNAs [6], but the methyltransferases that insert them have not been identified, and its functions have not been elucidated. Other modifications known to occur in eukaryotic mRNAs have either not been profiled in *E*. *coli* (as is the case for N1-methyladenosine, dihydrouridine, and N4-acetylcytidine) or failed to be detected (5-methylcytosine, [4]).

Pseudouridine is predominantly introduced into *E*. *coli* mRNAs by RluA, except for a few sites modified by RluD and RluC. RluA’s strong requirement for a UURNA motif, which is most often instantiated as UURAA, has deep implications for the distribution of pseudouridines in mRNAs. As a result of this sequence context, pseudouridines preferentially occur in valine (27% of high-confidence sites), leucine (24%), phenylalanine (20%), and isoleucine (13%) codons. In addition, the modification very rarely occurs in stop codons, if at all, as none of our high-confidence sites are located within a stop codon. Meanwhile, very little can be elucidated about the binding requirements for the synthases RluC and RluD from this dataset due to the small number of total sites. A single RluC-dependent mRNA site was found, which shares a GNΨG sequence motif with the three RluC-dependent rRNA sites (S3C Fig). The three RluD-dependent sites in mRNA sites are each directly preceded by a C, which is true of only one of its rRNA sites (S3B Fig). A previous study showed that purified RluD and RluC each modify many more sites *in vitro* than they do in cells [19], which suggests the participation of additional factors. Once introduced, the pseudouridine is unlikely to be removed from the transcript, as no enzymes with the capacity to reverse pseudouridylation have yet been identified. In this case, only as a result of mRNA turnover coupled with changes in pseudouridylation activity would the distribution of the modification in mRNAs change.

When binding its targets, RluA recognizes the target sequences by refolding them into a novel structure [36]. A crystal structure of RluA in complex with the anticodon stem-loop of one of its target tRNAs shows that the bound sequence is rearranged to flip out bases A_+2_ and N_+5_ (positions relative to the pseudouridine) and the target uridine (U_32_ in tRNA) while inducing positions U_+1_ and A_+4_ to form a reverse-Hoogsteen base pair [36]. These observations are consistent with the patterns we observe among mRNA targets: positions U_+1_ and A_+4_ are highly conserved among our sites, and the observed preference for the pseudouridines to occur in loops might facilitate this induced conformation.

Our results indicate that RluA potentially binds to the mRNA transcripts of dozens of genes, which may by itself have consequences for the target RNA in addition to the direct properties of pseudouridine. Some RNA-modifying enzymes function as RNA chaperones [40,41], unfolding the target RNA and providing the opportunity to re-fold into the correct conformation. This activity has been demonstrated for the pseudouridine synthase TruB in its interactions with tRNA. Loss of TruB results in misfolding of tRNAs and a defect in aminoacylation. However, expressing a catalytic null mutant of TruB, able to bind to its tRNA targets but unable to modify them, restores normal tRNA function [40]. It is possible that such a chaperone function may take place during mRNA modification by RluA, particularly if the modification occurs within a structured region, as we have demonstrated through analyzing RNA structure probing data.

We identified pseudouridylation of the 5′ UTR of *fimA* by RluA, as well as RluA-dependent expression in the *fim* operon. The *fim* operon encodes the components of the type 1 pilus [42]. This operon is highly regulated, combining regulation of its promoter, control of two different isoforms, and extensive secondary structure of its 5′ UTR. At the transcriptional level, expression of the operon can be turned on or off by recombinases that flip the upstream promoter, generating heterogeneity at this locus (*i*.*e*., phase variation) [35,43]. In pathogenic strains of *E*. *coli*, regulation of this operon is key for virulence and adhesion. Interestingly, expression of RluA is significantly upregulated during infection of bladder epithelial cells [44]. However, our experiments showed that *fim* upregulation in Δ*rluA* was not directly mediated by loss pseudouridylation in its 5′ UTR. Rather, it appears that the knockout results in more frequent inversion of the upstream regulatory element *fimS* into a conformation that increases transcription of the *fim* operon. How RluA may influence the frequency of phase variation remains to be determined, and the observed changes in *fimS* orientation could be either a direct or indirect effect of the loss of RluA

Notably, one gene outside of the *fim* operon was differentially expressed to a similar extent as *fimA* (Fig 4A). This gene, *pdeL*, encodes a cyclic-di-GMP phosphodiesterase and transcriptional regulator. In our data, *pdeL* is not expressed sufficiently in the wildtype to determine whether its mRNA is pseudouridylated. Until recently, PdeL was only known to regulate its own transcription, but now additional targets have been identified by ChIP [45]. Therefore, it will be important to determine whether *pdeL* carries out any regulation upstream or downstream of *fimA*, and whether pseudouridine plays a role in a regulatory network involving these genes.

Our results provide the first evidence for pseudouridine in bacterial mRNA. To identify phenotypes associated with these sites, it will be useful to survey growth and media conditions where loss of RluA confers a competitive disadvantage or growth defect, or where RluA is significantly differentially expressed. As with the example of uropathogenic *E*. *coli* infection, existing expression data from other virulent strains of *E*. *coli* may provide helpful leads. mRNA pseudouridylation by RluA family enzymes appears to be a highly conserved activity, as homologs RPUSD2 and RPUSD4 carry out modification of human pre-mRNAs [46].

## Materials and methods

### Strains

Pseudouridine synthase knockout strains were derived from the Keio collection [47]. Each knockout was transduced into an MG1655 background using P1 phage transduction, as described in [48]. Plate lysates were generated by growing each strain to mid-log phase in LB+0.1% glucose, adding CaCl_2_ to a final concentration of 5 mM, and mixing 3 mL of this culture with 50 μL of an existing P1_*vir*_ stock. This mix was allowed to adsorb for 20 minutes, then mixed with 5 mL of melted top agar and poured onto 2 plates (LB with 0.1% glucose, 2.5 mM CaCl_2_, 1.5% agar). The plates were incubated overnight, then top agar was scraped into 50-mL tubes and vortexed with 5 drops of chloroform. To store the new P1_*vir*_ stock, the tube was centrifuged for 10 minutes, and the supernatant transferred to a new tube to be stored at 4°C. To perform the P1 transduction, 1.5 mL of overnight cultures of *E*. *coli* MG1655 in LB were pelleted and resuspended in 0.75 mL P1 salts (10 mM CaCl_2_, 5mM MgSO_4_). 100 μL of the resuspended cells were mixed with 1–100 μL of the P1 lysate and left to adsorb at room temperature for 30 minutes. 1 mL of LB and 200 μL of 1 M sodium citrate were added to the mixture, then the cells were grown for 1 hour at 37°C. The cells were pelleted and resuspended in 100 μL LB, then spread on LB plates supplemented with 50 ng/mL of kanamycin.

### Growth conditions

Starter cultures were grown overnight in 5 mL EZ Rich MOPS complete media at 37°C. To start the experiment cultures, 1–2 μL of the overnight starter cultures were inoculated into 15 mL pre-warmed MOPS complete media, to OD_600_ < 0.001. These were incubated at 37°C shaking in glass flasks at 220 rpm until they reached a target OD_600_ between 0.10 and 0.15. This setup aimed to obtain exponential-phase *E*. *coli* cultures that had undergone 8 or more doublings since back-dilution.

### RNA extraction for RT-qPCR and BID-seq

For RT-qPCR and BID-seq, 7 mL culture was combined with 7 mL ice-cold methanol in 15 mL tubes, and pelleted and flash frozen. Pellets were resuspended in 100 μL Tris-EDTA pH 8.0 buffer with 10 mg/mL lysozyme and incubated at 37°C for 5 minutes to lyse cells. RNA was then depleted of gDNA and extracted using an RNEasy Mini Plus kit (Qiagen) according to manufacturer instructions.

### RT-qPCR

RNA was extracted as described above, then treated with TURBO DNase (Thermo Fisher) to remove residual genomic and plasmid DNA by combining 10 μg of RNA with 5 μL TURBO DNase and 1 μL buffer and bringing to a final volume of 50 μL. DNase reactions were incubated at 37°C for 30 minutes, then subsequently inactivated by adding 8.8 μL 100 mM EDTA and incubating for an additional 10 minutes at 75°C, and cleaned up with an RNA Clean and Concentrate-25 kit (Zymo) according to manufacturer instructions. 2 μg RNA was reverse transcribed with SuperScript III (Thermo Fisher) and random hexamer priming (5 μM final concentration) for 1 hour at 50°C. RNA was then degraded by adding 10 μL 1M NaOH, heating at 95°C for 5 min, and neutralizing with 10 μL 1 M HCl, after which samples were brought to 200 μL. qPCR was performed with 10 μL KAPA SYBR qPCR Master Mix, 4 μL cDNA, and 3 μL of each primer on a Light Cycler 480 II Real-Time qPCR Machine. *fimA-gfp* levels were measured with primers in GFP (oHL205/206) and normalized to expression of *hcaT* (oJD070/071) [49]. Uncertainty was calculated as propagated standard error of the mean. Control measurements from wild type *E*. *coli* not carrying the reporter plasmid were 7 or more Ct values greater than measurements in reporter-carrying strains.

### RNA extraction for Pseudo-Seq and Rend-Seq

RNA samples to be used for Pseudo-Seq and Rend-Seq were extracted following the RNA snap protocol [50]. Briefly, cultures were transferred to 15-mL tubes and pelleted by centrifugation at 3220 ×*g*, at 4°C. The media was decanted, then the tubes were spun down for an additional minute. The remaining media was aspirated and the pellets were flash frozen in liquid nitrogen. For RNA extraction, 200 μL of RNA extraction solution (consisting of 18 mM EDTA, 0.025% SDS, 1% 2-mercaptoethanol, 95% formamide) were added to each pellet, followed by vortexing for 20 seconds at top speed. The mixture was transferred to 1.5-mL tubes and incubated at 95°C for 7 minutes in a ThermoMixer. To remove cell debris, tubes were centrifuged at >18000 ×*g* at 4°C for 5 minutes. Extracted RNA was precipitated in ethanol by mixing 50 μL of the supernatant with 200 μL of 10 mM Tris (pH 7.0), 75 μL of 3 M sodium acetate (pH 5.3) and 825 μL of ethanol.

### RNA purification by size selection

During library preparation, several steps will call for the purification of RNA by size selection using excision from polyacrylamide gels. RNA pellets are resuspended in 6 μL of 10 mM Tris pH 7, or similarly small volume appropriate for loading into gel wells. A 10% TBE-Urea gel is pre-run at 200V (or appropriate voltage) for at least 30 minutes. The samples are prepared by adding 1 volume of 2X TBE-Urea Loading Dye, denaturing at 80°C for 10 minutes, and finally placing back on ice. Samples are loaded into the gel wells, and the gel is run at 200V (or appropriate voltage) until the dye fronts indicate that the desired separation has been achieved, which will vary depending on the size of fragment to be excised. To visualize the RNA bands, the gel was soaked in SYBR gold diluted 1:10000 in 10 mM Tris pH 7, then lit on a SafeImager. Gel fragments were cut out and transferred to a 1.5-mL tube with 400 μL 10 mM Tris pH 7 and eluted overnight at 4°C. Alternatively, the gel fragments may be crushed and eluted by incubating at 80°C, shaking for 10 minutes. The solvent is transferred to a new tube and precipitated.

### Pseudo-Seq library preparation

Pseudo-Seq protocol was based on [1,16], with some adjustments for handling of the shorter transcripts in *E*. *coli*. Total RNA (see RNA Extraction section above) was purified using the QIAGEN RNEasy Plus Mini Kit, which depletes RNAs shorter than 200 nt and removes genomic DNA. rRNA was depleted using either RiboZero or MicrobExpress kits. RNA was fragmented by adding 4.4 μL of 10X RNA fragmentation buffer (ThermoFisher), incubating at 95°C for 22 seconds, then quenched with 5uM of stop solution (ThermoFisher). CMC treatment was performed by incubating RNA in 40 μL of 5 mM EDTA at 80°C for 3 minutes, then adding 100 μL of 0.5 M CMC in BEU buffer (7 M urea, 4 mM EDTA, 50mM bicine, then incubating at 40°C for 45 minutes, shaking at 1000 rpm. Mock treatment was performed by adding 100 μL BEU without CMC before incubating. RNAs were precipitated in isopropanol and resuspended in 30 μL of pH 10.4 buffer (50 mM Na_2_CO_3_, 2mM EDTA, adjusted to pH 10.4), then incubated at 50°C for 2 hours to reverse CMC adducts from guanidines and uridines. RNA fragments were precipitated, then run on a 10% TBE-Urea gel, and fragments 100–115 nt long were excised and purified. RNA 3′ ends were dephosphorylated with T4 polynucleotide kinase (PNK), at 37°C for 1 hour. Enzyme was inactivated at 75°C for 10 minutes. 3′ adapters were ligated onto RNA fragments using T4 RNA ligase 2 truncated (20 μL reaction with ∼3 pmols input RNA, 25% PEG, 5 μM Linker-1 [5′ App/CTGTAGGCACCATCAAT/3ddC]) for 2.5 hours at 25°C. Ligated RNAs were purified by size selection. Reverse transcription (RT) was carried out with SuperScriptIII: adapter-ligated RNAs were annealed to the RT primer (oCSB76, CAAGCAGAAGACGGCATACGAGATATTGATGGTGCCTACAG) in 11 μL of water with 25 pmol of the RT primer, incubated at 65°C for 5 minutes. Reverse transcription proceeded at 50°C for 30 minutes, with 0.5 mM dNTPs, 5 mM DTT, 10 U/μL SuperScriptIII and 1 U/μL SuperaseIn. NaOH was added to a final concentration of 0.1M, and the mixture was incubated at 95°C for 20 minutes to hydrolyze template RNA. This solution was mixed with 20 μL of 2X RNA loading dye, and loaded onto a 10% TBE-Urea gel. Products 66–116 nt long, corresponding to insert sizes of 25–75 nt, were excised and purified. An adapter (oCSB77, /5Phos/NNNNNNNNNNAGATCGGAAGAGCGTCGTGTAGGGAAAGAGTGT/3SpC3/) was ligated to the 3′ ends of cDNAs as follows: cDNA was incubated for 2 minutes at 75°C with 8pmols of the adapter and 1 μL of DMSO in a reaction volume of 10.8 μL. Then the rest of the ligation components were added (final reaction volume of 23 μL, 0.9 mM ATP, 20% PEG-8000, 1 μL T4 RNA ligase, high concentration), and the reaction proceeded overnight at 25°C. Partway through the incubation, the reactions were supplemented with an additional 0.5 μL of ligase. The reactions were precipitated, and ligated cDNAs were purified by size selection. After PCR amplification, libraries were sequenced on an Illumina NextSeq, generated 75-nt single-end libraries.

### REnd-Seq library preparation

REnd-Seq was performed as described in [51]. rRNA was depleted from 10 μg total RNA (see RNA Extraction section above) with MICROBexpress, using two reaction volumes. Reaction was precipitated in isopropanol. RNA was fragmented using RNA Fragmentation reagents, by first incubating RNA in 40 μL water at 95°C for 2 minutes, then adding Fragmentation Buffer to 1X and incubating for 25 seconds at 95°C and finally quenching with Stop Buffer. Samples were precipitated in isopropanol and resuspended in 5 μL of 10 mM Tris pH 7. To size-select, RNA was mixed with one volume of 2X TBE-Urea sample loading buffer and run on a 15% TBE-Urea gel. RNA fragments 15–45 long were excised and purified, then precipitated. RNA was dephosphorylated with T4 polynucleotide kinase (PNK) in a 20-μL reaction, incubated at 37°C for 1 hour, then inactivated at 75°C for 10 minutes. RNA was precipitated in isopropanol. Linker-1 was ligated to 3 moles RNA 3′ ends using T4 RNA ligase 2 (truncated), in a 20-μL reaction containing 25% PEG-8000, 1X T4 RNA ligase buffer, and 100 pmol of Linker-1. Reactions were precipitated in isopropanol and resuspended in 5 μL 10 mM Tris pH 7. To purify ligated RNA, samples were mixed with one volume of 2X loading dye and ran on a 10% TBE-Urea gel. Ligated fragments were excised, purified, and precipitated. Reverse transcriptions were carried out with SuperScript III. First, samples were incubated with 25 pmol of RT primer (oCJ485) in 12 μL water at 65°C for 5 minutes, then placed on ice. Remaining reaction reagents were added to the sample for a final volume of 20 μL (1X First Strand Buffer, 0.5 mM dNTPs, 5 mM DTT, 20U SuperaseIn and 1 μL SuperScriptIII), and incubated at 50°C for 45 minutes. NaOH was added to a final concentration of 0.1M, and the mixture was incubated at 95°C for 20 minutes to hydrolyze template RNA. This solution was mixed with 20 μL of 2X RNA loading dye, and loaded onto a 10% TBE-Urea gel. cDNAs were excised, purified, and precipitated.

### Pseudo-Seq data analysis

Adapter sequences were trimmed from read 3′ ends using bbduk (parameters: ktrim = r k = 10 hdist = 1 mink = 1). 10-nt unique molecular identifiers (UMI) were removed from the 5′ end of the reads and added to the record headers using a custom script. Reads were mapped to the *E*. *coli* MG1655 genome (NC_000913_3) using bbmap (slow = t perfectmode = true k = 11 ambiguous = all). Alignments were converted to bam files and sorted with samtools, then filtered to keep only uniquely mapping reads. Multi-mapping reads were parsed into a separate bam file. PCR duplicates were collapsed by means of the UMI, using a custom script. Read 5′ ends were quantified on each strand using bedtools (bedtools genomecov -d -5 -strand +), and were then saved as wig files.

### Peak calling

We calculate a Z-score for each U position relative to the 100-nt window surrounding it, requiring that at least 100 reads map to the window. Before the Z-score calculation, we modify the background window in two ways. First, we determine whether the window spans a strong transcript end. If it does, we shift the background window to avoid the peaks to achieve the following criteria: the 5′ boundary of a window must be at least 40-nucleotides downstream of the nearest transcript 5′ end, and at least 5 nucleotides downstream of the nearest transcript 3′ end. On the other side, the 3′ boundary of a window must be at least 25 nt upstream of the nearest downstream 3′ end, and at least 5 nt upstream of the nearest downstream transcript 5′ end. These adjustments are implemented to minimize the impact of read coverage biases close to transcript ends, and from differences in coverage between overlapping transcript isoforms. Transcript end positions were obtained from wild-type REnd-Seq data in the same growth conditions. To reduce the impact of nearby outliers (such as peaks from other modifications and secondary structure) on Z-score calculation, we apply 95% winsorization to the window surrounding the target site, in which all positions whose normalized read count was greater than the 95^th^ percentile for the window were adjusted to be equal to the 95^th^ percentile. The value of the target site itself is not changed.

Z-scores are calculated relative to the adjusted 100-nt background window. To call CMC-dependent peaks, we require a Z-score ≥ 15 in the CMC-treated library, and for the CMC-treated Z-score to be at least 3-fold greater than the corresponding Z-score in the mock-treated library (Fig 2B). A CMC-dependent site is assigned as dependent on a given pseudouridine synthase if it meets three criteria: (1) it has sufficient coverage in the knockout library, (2) the knockout peak Z-score < 10, and (3) the WT CMC Z-score is at least 4-fold greater than the knockout peak.

### Targeted Bisulfite Sequencing (BID-Seq)

Total RNA was prepared for bisulfite sequencing using a method adapted from [33]. Specifically, 8.5 μL of RNA (1 μg total) was combined with 45 μL 2.4M Na_2_SO_3_, 0.36M NaHSO_3_, and heated at 70°C for 3 hours. RNA was brought to 100 μL with RNAse-free water and desalted on a PD MiniTrap G-25 spin column. RNA was desulfonated by adding 100 μL 2M Tris-HCl (pH 9) and heated at 37°C for 2 hours. RNA was recovered with ethanol precipitation and resuspended in 5 μL RNase-free water. For reverse-transcription, random-hexamer primers were annealed to RNA by combining 5 μL RNA, 1 μL 50 mM random-hexamers, 10 mM dNTPs, 6 μL water and heating at 65°C for 5 minutes, then snap-chilling on ice for 1 minute. Primer-annealed RNA was combined with 4 μL 5x SuperScript IV buffer, 100 mM DTT, 1 μL RNaseIn Plus, 1 μL SuperScript IV, and incubated at 23°C for 10 minutes, 55°C 10 minutes, 80°C 10 minutes. RNA was hydrolyzed by addition of 5 μL 1M NaOH and heating at 95°C for 5 minutes, then quenching with 5 μL 1M HCl. Targeted primers were used to PCR amplify each target site, which were purified using Ampure-XP beads and pooled at equal concentration before submission for amplicon sequencing (Genewiz). Raw fastq files were aligned to a reference of target genes using bbmap, and the resulting bam files sorted and indexed using samtools. Deletion rate at each position was quantified using the samtools mpileup command and custom python scripts.

### Structure analysis

In-cell DMS-Seq reactivities for wild-type *E*. *coli* were obtained from [37]. Coordinates were converted to NC_000913_3 using LiftOver. For each site of interest, reactivities for the surrounding 100-nt window were parsed from this dataset into individual files (since only As and Cs react with DMS, other bases had a placeholder value of -999). These were provided as input to RNAfold for DMS-informed secondary structure prediction, using the ‐‐shape option (with parameters -p, -T 37, ‐‐shapeMethod = Z). Pairing probability matrices were parsed from the RNAfold output files, and columns were summed to summarize pairing probability. Pairing probabilities were visualized by calculating relative positions relative to the site of interest and visualizing the pairing probability distributions for groups of sites at each relative position.

### Background site selection

A set of background sites was selected to control for expression level and for the influence of the RluA motif. First, we used our REnd-Seq data set to obtain WT expression levels for each gene that contains a high-confidence Ψ site. We consider only uridine positions within genes with RPKM ≥ 105 in our wild-type dataset, which was the lowest expression level among the genes containing high-confidence sites. Within these genes, we search for instances of the UURAA motif, and remove any sites that are within 100-nt of a Ψ site.

Secondary structure diagrams were generated using VARNA (v3.93)

## Supporting information

S1 Fig Validation of known sites in three tRNAs: tRNA-Cys(GCA) (top), tRNA-His(GUG) (middle), and tRNA-Pro(GGG) (bottom). PseudoSeq data tracks for CMC-treated (black) and mock-treated (gray) samples. Pseudouridine position is indicated with triangles on the secondary structure, and with blue dashed lines on the data track. The top panel illustrates an instance of nearby modifications masking each other, where the strong peak at Ψ39 hinders detection of Ψ32 due to being encountered by the reverse-transcriptase first, thus generating a larger population of fragments truncated at Ψ39 than at Ψ32. Having multiple modifications in very close proximity makes this a particular issue with detection in tRNAs.
(EPS)

S2 Fig Z-score calculation before and after winsorization. (a) Conceptual example illustrating background winsorization. (left) Normalization window for a pseudouridine site with strong neighboring peaks generated from nearby modifications, as is common in rRNA. (right) 95th percentile winsorization is applied to the same background window, reducing the value of the nearby outliers, but not of the target site itself, to the value of the 95th percentile. For (b) and (c) Z-scores for reference U-sites are shown and compared between a wild-type CMC-treated library and a mock-treated library. Known pseudouridine sites are colored in orange, other modified uridines are in blue, and unmodified uridines are in gray. Panel (b) shows Z-scores calculated without winsorization and panel (c) shows Z-scores for the same data calculated with winsorization. The two pseudouridines with Z-score < 15 are Ψ1911 and Ψ2604 (Z-score = 3.72 and 7.25, respectively). Detection of Ψ1911 is hindered by its proximity to two downstream modifications, m3Ψ1915 and Ψ1917, as reverse-transcriptase encounters these events before Ψ1911, generating a larger population of fragments truncated at the downstream events. Similarly, Ψ2604 is directly upstream of another pseudouridine site, Ψ2605. (d) Selection of coverage cutoff. At high confidence sites, reads from a CMC-treated library (orange) and a mock-treated library (gray) were subsampled to various levels (x-axis shows the total number of reads mapping to the window after subsampling) and the corresponding Z-score was calculated (y-axis). Coverage cutoff of ≥ 100 reads is shown as dashed lines. (e) Expression level distributions for all expressed genes, genes that met coverage cutoffs in the wild-type Pseudo-Seq library, and genes containing a pseudouridine site. Gene counts are annotated at the top
(EPS)

S3 Fig Reproducible RluC- and RluD-dependent sites in mRNA. (a) PseudoSeq signal in the *atpG* mRNA for CMC-treated wild-type and CMC-treated knockouts of RluA, RluC, and RluD (orange) and for mock-treated (gray). The site appears as a RluC- and CMC-dependent peak in read 5′ ends. (b) Sequence alignment of RluD-dependent rRNA (gray) and mRNA (black) sites. (c) Sequence alignment of RluC-dependent rRNA and mRNA sites, with common nucleotides in bold.
(EPS)

S4 Fig Validation of mRNA pseudouridylation events using BID-Seq. Deletion traces of all sites sequenced by targeted bisulfite-sequencing, used for the bar plot in Fig 3D. Plots show the deletion rate at each relative position in the gene. Deletions are generally 1–2 nt long.
(EPS)

S5 Fig Pseudouridylation by RluA does not affect expression level of a constitutively expressed *fimA-gfp* reporter transcript. (a) The reporter construct consists of the *fimA* 5′ UTR and *fimA* gene fused to superfolder GFP, expressed from a constitutive promoter that is distinct from the *fim* promoter. (b) Reporter expression was measured by RT-qPCR with *hcaT* as a reference gene.
(EPS)

S6 Fig The UURNA motif is necessary but not sufficient for RluA-dependent pseudouridylation. (a) Pseudo-Seq signal (Z-score) for all uridines that match the RluA motif, colored by sequence (UURAA in dark blue, UURBA in green, and UUYAA in light blue, where R = purines, Y = pyrimidines and B = not A) in CMC-treated wild-type (y-axis) vs ΔrluA (x-axis) samples. Only uridines in protein-coding genes are included in this analysis. (b) Z-score distribution (complementary cumulative density function) for these positions.
(EPS)

S7 Fig Predicted structures of additional RluA-dependent sites. DMS-informed secondary structure predictions for all RluA-dependent sites not shown in Fig 4, carried out in the same way. White-to-green gradient indicates DMS reactivity. Non-reactive nucleotides are in gray.
(EPS)

S1 Table Peak heights across all libraries for sites meeting the coverage cutoff. Event annotations and enzyme assignments are also included.
(CSV)

S2 Table Summary of events detected by simulating higher read coverage. For each event, peak heights are reported for the top depth where it was detected.
(CSV)
